# Expression of osteoprotegerin and its ligands, RANKL and TRAIL, in rheumatoid arthritis

**DOI:** 10.1038/srep29713

**Published:** 2016-07-12

**Authors:** Sara Remuzgo-Martínez, Fernanda Genre, Raquel López-Mejías, Begoña Ubilla, Verónica Mijares, Trinitario Pina, Alfonso Corrales, Ricardo Blanco, Javier Martín, Javier Llorca, Miguel A. González-Gay

**Affiliations:** 1 Epidemiology, Genetics and Atherosclerosis Research Group on Systemic Inflammatory Diseases, IDIVAL, Santander, Spain; 2 Institute of Parasitology and Biomedicine López-Neyra, IPBLN-CSIC, Granada, Spain; 3 Department of Epidemiology and Computational Biology, School of Medicine, University of Cantabria, and CIBER Epidemiología y Salud Pública (CIBERESP), IDIVAL, Santander, Spain; 4 School of Medicine, University of Cantabria, Santander, Spain

## Abstract

Osteoprotegerin (OPG), receptor activator of nuclear factor-ΚB ligand (RANKL) and tumor necrosis factor-related apoptosis-inducing ligand (TRAIL) have been involved in rheumatoid arthritis (RA) pathophysiology. In this study, we assessed messenger RNA (mRNA) expression of these molecules by qPCR in peripheral blood from 26 patients with RA (12 of them with ischemic heart disease –IHD) and 10 healthy controls. Correlation coefficients between *OPG, RANKL* and *TRAIL* expression levels in RA patients and their clinical and demographic characteristics were also evaluated. Whereas *OPG* and *OPG/TRAIL* ratio expression were significantly increased in RA patients compared to controls (fold change = 1.79, p = 0.013 and 2.07, p = 0.030, respectively), *RANKL/OPG* ratio was significantly decreased (fold change = 0.50, p = 0.020). No significant differences were found between patients and controls in *RANKL* and *TRAIL* expression. Interestingly, *TRAIL* expression was significantly higher in RA patients with IHD compared to those without IHD (fold change = 1.46, p = 0.033). Moreover, biologic disease-modifying antirheumatic drugs (DMARDs) significantly decreased *RANKL* expression in RA patients (p = 0.016). Our study supports an important role of *OPG* and *TRAIL* in RA. Furthermore, it highlights an effect of biologic DMARDs in the modulation of *RANKL*.

Osteoprotegerin (OPG)/receptor activator of nuclear factor-ΚB ligand (RANKL)/tumor necrosis factor (TNF)-related apoptosis-inducing ligand (TRAIL) system has been involved in the pathophysiology of rheumatoid arthritis (RA)^1^,^2^. OPG is a soluble glycoprotein, member of the TNF-receptor superfamily and an inhibitor of two cytokines belonging to the TNF ligand family, RANKL and TRAIL^3^. RANKL functions as a key factor for osteoclastogenesis, since the binding with its receptor, receptor activator of nuclear factor-ΚB (RANK), favors the activation of osteoclasts and bone resorption. OPG reduces RANKL-RANK interactions and thus inhibits osteoclastogenesis^3^. In this regard, RANKL/OPG ratio plays an important role regulating bone homeostasis in RA, a disease characterized by bone and cartilage destruction^1^,^4^. Furthermore, a protective role in RA has been shown for TRAIL, an anti-inflammatory molecule mainly known as inductor of apoptosis in tumor cells. Thus, TRAIL could be involved in the regulation of the systemic inflammatory autoimmune response produced by the disease^2^. However, increasing evidence suggest it could be a pleiotropic cytokine with a dual function^5^. Since OPG can bind to TRAIL inhibiting their function, it has been proposed that the OPG/TRAIL ratio could also be involved in RA pathogenesis^6^.

It is well known that RA patients have an accelerated atherosclerotic process, leading to an increased risk of cardiovascular (CV) disease, including ischemic heart disease (IHD)^7^. In this sense, OPG/RANKL/TRAIL system has also been related to atherosclerosis and CV disease^8^. Due to the important role that these molecules play in CV disease, they have been proposed as potential biomarkers of CV disease^8^,^9^.

Previously, our group reported that OPG serum levels were associated with CV disease in RA patients^10^. Therefore, to confirm the role of OPG and its ligands, RANKL and TRAIL, in the pathophysiology of RA, we determined for the first time the differential gene expression of these molecules in peripheral blood from patients with RA, with and without IHD, and healthy controls. Changes in expression level of these molecules and its correlation with clinical and demographic characteristics of RA patients will also be useful to elucidate this role.

Taking into account that both RA and CV disease are inflammatory systemic diseases, the assessment of *OPG, RANKL* and *TRAIL* expression at a systemic level (blood) is of main importance.

## Patients and Methods

### Patients and controls

For experiments involving humans and the use of human blood samples, all the methods were carried out in accordance with the approved guidelines and regulations, according to the Declaration of Helsinki. All experimental protocols were approved by the Ethics Committee of clinical research of Cantabria (CEIC-C, Number of reference 14/2012). Informed consent was obtained from all subjects.

Twenty-six RA patients, 12 of them with IHD, and ten healthy controls matched for age and sex were recruited from Hospital Universitario Marqués de Valdecilla (Santander, Cantabria, Spain). All RA patients met the 2010 American College of Rheumatology classification criteria for RA^11^. We recorded the main demographic and clinical characteristics of patients, measured the erythrocyte sedimentation rate (ESR), C-reactive protein (CRP) and lipids, and calculated the Disease Activity Score in 28 joints (DAS28)-CRP and –ESR at the time of assessment. The presence of IHD and traditional CV risk factors were defined as previously reported^10^. The characteristics of the RA patients and controls included in this study have previously been described^12^.

### Blood RNA extraction

As we previously described, total RNA was isolated from peripheral blood samples according to the manufacturer’s protocol using NucleoSpin RNA Blood Midi Kit (Macherey-Nagel)^12^. Samples were concentrated using GeneJET RNA Cleanup and Concentration Micro Kit (Thermo Scientific) and stored at −80 °C until further processing.

### Quantitative real-time polymerase chain reaction (PCR)

For each sample, 1 μg of total RNA was reverse transcribed into cDNA using iScriptTM Advanced cDNA Synthesis Kit for RT-qPCR (Bio-Rad, Hercules, CA, USA). 20 ng of cDNA was used for quantitative real-time PCR (qPCR), using SsoAdvancedTM Universal SYBR^®^ Green Supermix (Bio-Rad, Hercules, CA, USA). Primers for amplification of *OPG*, *RANKL*, *TRAIL*, *beta-actin* and *GAPDH* were acquired from PrimePCR Assays, Bio-Rad, Hercules, CA, USA. All samples were assayed in triplicate and controls were included in each reaction. qPCRs were performed in a 7900 HT real-time instrument (Applied Biosystems, Foster City, CA, USA) using the following conditions: stage 1: 95 °C for 2 min, stage 2: 95 °C for 5 sec followed by 60 °C for 30 sec repeated for 45 cycles. The threshold cycle (Ct) was manually established and recorded by the SDS 2.2.2 software (Applied Biosystems, Foster City, CA, USA). The relative *OPG, RANKL* and *TRAIL* messenger RNA (mRNA) expression was analyzed by the comparative Ct method, using *beta-actin* and *GAPDH* as housekeeping genes^12^. Normalized expression levels were obtained for each sample and the *RANKL/OPG* and *OPG/TRAIL* ratios were calculated with these data. Mean values were determined for each study group and fold-change (changed amount of expression) was calculated by comparing the means between different groups. A fold-change value less than 1 indicates negative or down-regulation of the study gene. A fold-change value greater than 1 indicates positive or up-regulation.

### OPG, RANKL and TRAIL serum levels by enzyme-linked immunosorbent assay (ELISA)

OPG levels were measured as previously described^10^. Concentrations of free soluble RANKL and TRAIL were also determined by ELISA, using kits from Biomedica (BI-20462) and R&D Systems (DTRL00), respectively, according to the manufacturer’s instructions. All samples were analyzed in duplicate.

### Statistical analysis

Results were expressed as mean ± standard deviation (SD). Normalized expression values and protein levels obtained for each sample were analyzed by GraphPad Prism^®^ 3.0 (GraphPad Software, San Diego, CA, USA) using nonparametric Mann-Withney U test to compare two study groups. A p value < 0.05 was considered statistically significant.

*OPG, RANKL* and *TRAIL* expression among patients with RA and controls was first compared by analysis of covariance (ANCOVA), with adjustment for age, sex, and traditional CV risk factors.

Partial correlations of demographic and clinical characteristics in RA patients with their relative *OPG*, *RANKL* and *TRAIL* mRNA levels were performed after adjusting for age at the time of the study, sex, and classic CV risk factors via estimation of the Pearson partial correlation coefficient (r). The same statistical analysis was conducted to analyze whether *OPG* expression levels were associated with *RANKL* and *TRAIL* mRNA expression. A p value < 0.05 was considered statistically significant. These analyses were performed using Stata 12/SE (StataCorp, College Station, TX).

## Results

### *OPG, RANKL* and *TRAIL* expression in RA

**Figure 1:**
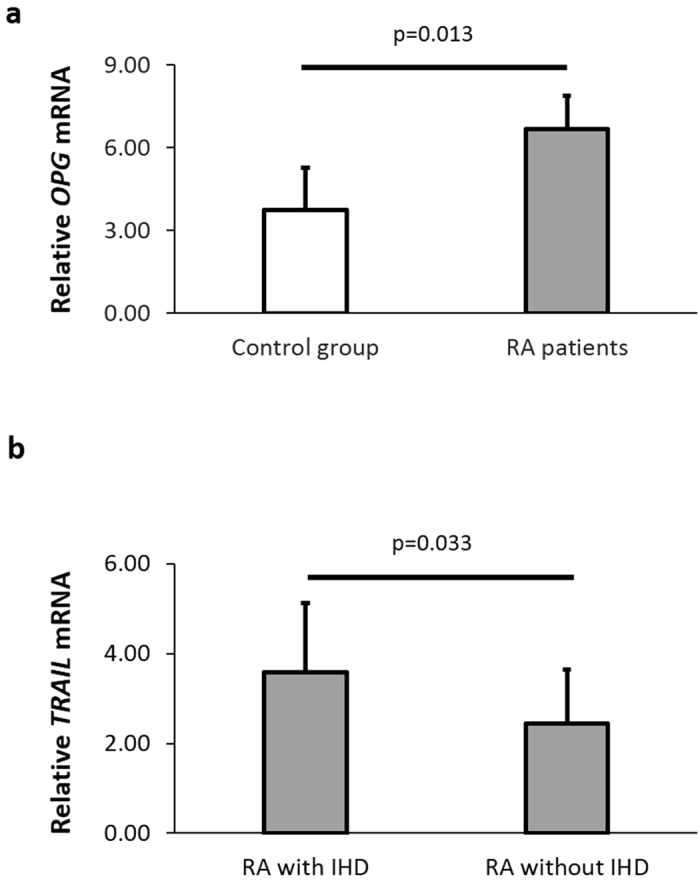
Increased *OPG* mRNA expression in patients with RA and *TRAIL* mRNA expression in RA patients with IHD. *OPG* and *TRAIL* expression was normalized to two housekeeping genes (*beta-actin* and *GAPDH*). (**a**) Differential expression of relative *OPG* mRNA was analyzed between control group (n = 10) and RA patients (n = 26). (**b**) Differential expression of relative *TRAIL* mRNA was analyzed between RA patients stratified according to the presence (n = 12) or absence of IHD (n = 14). Each bar represents mean value ± SD obtained for each sample in triplicate.

Expression of the *OPG* gene was significantly increased in RA patients compared to controls (fold change = 1.79, p = 0.013, Table 1). In this regard, patients with RA had a mean *OPG* expression of 6.68 ± 3.69 whereas controls had a mean *OPG* expression of 3.74 ± 2.00 (Table 1 and Fig. 1a). However, no significant differences were found between patients and controls in *RANKL* and *TRAIL* mRNA expression (Table 1). A decreased *RANKL/OPG* ratio and an increased *OPG/TRAIL* ratio was observed in patients compared to controls (fold change = 0.50 and 2.07, respectively). These differences in expression were statistically significant (p = 0.020 and p = 0.030, respectively, Table 1).

When RA patients were stratified according to the presence or absence of IHD, we disclosed that the relative *TRAIL* mRNA expression was significantly higher in RA patients with compared to those without IHD (3.58 ± 1.55 vs 2.45 ± 1.19, fold change = 1.46, p = 0.033, Fig. 1b). However, no significant differences were found in *OPG* and *RANKL* mRNA levels or in *RANKL/OPG* and *OPG/TRAIL* ratios between these two groups (data not shown).

### Association of *OPG, RANKL* and *TRAIL* mRNA expression with clinical and demographic characteristics in RA patients

An inverse association was found between *OPG* expression and total cholesterol (r = −0.48, p = 0.040). Additionally, a negative correlation was observed between *RANKL* expression and CRP (r = −0.52, p = 0.024) and DAS28 CRP (r = −0.56, p = 0.014). Interestingly, RA patients undergoing treatment with biologic disease-modifying antirheumatic drugs (DMARDs) showed a statistically significant decrease of *RANKL* expression (r = −0.54, p = 0.016). Regarding *TRAIL*, we disclosed a positive correlation with RA disease duration (r = 0.46, p = 0.047) and CRP (r = 0.58, p = 0.010).

### Influence of *OPG* expression on its ligands

No association was found between relative *OPG* mRNA expression and *RANKL* or *TRAIL* mRNA levels (p = 0.89 and p = 0.85, respectively) in RA patients.

### OPG, RANKL and TRAIL serum levels in RA

ELISA was performed in order to provide supplementary information on the *OPG*, *RANKL* and *TRAIL* mRNA expression and the corresponding protein levels. OPG serum levels were significantly increased in RA patients compared to controls (9.06 ± 6.00 vs 3.85 ± 1.51 ng/ml, p = 0.002). No significant differences were found between patients and controls in RANKL and TRAIL concentrations.

## Discussion

Several molecules have been implicated in the pathogenesis of both RA and CV disease^13^. In this regard, the OPG/RANKL/TRAIL system has been linked to bone loss in RA and CV risk^1^,^6^,^8^. To further study the involvement of this system in RA at the gene expression level, we evaluated the *OPG, RANKL* and *TRAIL* mRNA levels in peripheral blood from patients with RA and their relationship with clinical and demographical characteristics of RA disease. Furthermore, we studied its potential association with IHD in patients with RA.

Previously, it has been reported that the expression of many cytokines and growth factors is up- or down-regulated in both peripheral blood and local joints in patients with RA^14^. In this regard, circulating (or peripheral) blood cells from RA patients have been used in previous studies to assess the *OPG/RANKL/TRAIL* gene expression for prognosis or diagnosis of RA^14^,^15^. In addition, considering that RA and CV disease affect multiple tissues at a systemic level, we believe that the assessment of the role of these molecules in circulation is very relevant.

OPG regulates bone homeostasis upon binding to RANKL and also participates in the pathogenesis of atherosclerosis and CV diseases^8^. We previously described that OPG concentrations are independently associated with endothelial activation, carotid atherosclerosis and established CV disease in RA^10^,^16^. In the present study, we observed that the expression of *OPG* was up-regulated in RA patients, supporting its pivotal role in the pathogenesis of RA. Moreover, the increase in OPG serum levels disclosed in our patients with RA enables us to confirm that the upregulation of *OPG* at a systemic level leads to a high release of OPG protein in serum. In addition, *OPG* mRNA levels were inversely associated with total cholesterol. This finding is in line with former results of our group performed in RA and ankylosing spondylitis patients, which disclosed a negative correlation between OPG and total and LDL cholesterol, even if they were assessed regarding serum/plasma concentrations^16^,^17^. However, in another study from our group we could not find an association between OPG levels and cholesterol from a different cohort of patients with RA^10^. These results may appear to be contradictory at first glance. However, we believe that potential associations observed between a clinical characteristic or disease with gene expression at mRNA level does not necessarily have to be reflected in protein concentrations. Nevertheless, further studies with a larger number of samples should be performed to elucidate these discrepancies.

Regarding *RANKL* mRNA expression, no significant differences were found between our patients and controls. The same finding was previously shown in synovial fluid-neutrophils from RA patients compared to normal blood-neutrophils from healthy individuals^18^. In addition, we disclosed a negative association between *RANKL* mRNA and treatment with biologic DMARDs. Therefore, it is plausible to think that *RANKL* expression is being modulated at a transcriptional level by these drugs. Accordingly, biologic and synthetic DMARDs also decreased *RANKL* mRNA in fibroblasts from RA patients^19^,^20^.

TRAIL is another molecule involved in this system. It has also been associated with atherosclerosis and CV disease^2^,^8^. With respect to this, we disclosed a significant up-regulation of this gene in patients with IHD compared to those without IHD. In keeping with this result, TRAIL concentrations were found to be increased in RA patients with heart failure^21^. Since TRAIL is an anti-inflammatory molecule, the high expression observed in RA patients with IHD could be the result of a compensatory mechanism to limit the potential pro-inflammatory effects triggered by other cytokines. However, depending on the context, the role of TRAIL is still confusing^5^. Consequently, our data suggest that high expression of *TRAIL* may be a potential predictor of CV disease in RA patients. In addition, the positive relationship between *TRAIL* mRNA expression and both CRP concentrations and RA disease duration reinforces our hypothesis. In this regard, CRP, involved in systemic inflammation and CV disease, has also been related to TRAIL serum levels in other chronic inflammatory arthritis, such as psoriatic arthritis^22^.

Finally, the upregulation of *OPG* observed in our RA patients led to an increased *OPG/TRAIL* ratio. Since *TRAIL* mRNA was associated with IHD in our study, this ratio may also play an important role in the development CV disease. This finding is agreement with Secchiero *et al*., who disclosed an increased in OPG/TRAIL ratio levels in patients with acute myocardial infarction who developed heart failure^23^.

Our study supports an important role of *OPG* and *TRAIL* in RA. Furthermore, it highlights an effect of biologic DMARDs in the modulation of *RANKL*.

## Additional Information

**How to cite this article**: Remuzgo-Martínez, S. *et al*. Expression of osteoprotegerin and its ligands, RANKL and TRAIL, in rheumatoid arthritis. *Sci. Rep.* **6**, 29713; doi: 10.1038/srep29713 (2016).

## Figures and Tables

**Table 1:** *OPG, RANKL, TRAIL* and *RANKL/OPG* and *OPG/TRAIL* ratios mRNA expression in controls and RA patients.

mRNA expression (normalized to *beta-actin* and *GAPDH* genes)	Controls n = 10	RA patients n = 26	p value
*OPG*	3.74 ± 2.00	6.68 ± 3.69	**0.013**
*RANKL*	3.67 ± 0.91	3.48 ± 1.41	0.68
*TRAIL*	3.20 ± 1.30	2.97 ± 1.46	0.51
*RANKL/OPG* ratio	1.33 ± 0.95	0.67 ± 0.42	**0.020**
*OPG/TRAIL* ratio	1.36 ± 0.77	2.81 ± 2.18	**0.030**

Values are given as mean ± SD. Significant results are highlighted in bold (p < 0.05).
